# Aminoguanidine attenuates arsenic-induced hepatic oxidative stress: Dose-dependent effects in a mouse model

**DOI:** 10.1016/j.toxrep.2025.102136

**Published:** 2025-10-09

**Authors:** Behnam Ghorbani-Nejad, Matin Baghani, Nastaran Allahdini Hasaruyieh, Mahshid Jamshidi, Leila Poudineh, Somayyeh Karami-Mohajeri, Milad Rahimzadegan, Jafar Ahmadi

**Affiliations:** a Department of Pharmacology & Toxicology, School of Pharmacy, Kerman University of Medical Sciences, Kerman, Iran; b Pharmaceutics Research Center, Institute of Neuropharmacology, Kerman University of Medical Sciences, Kerman, Iran; c Student Research Committee, Kerman University of Medical Sciences, Kerman, Iran; d Functional Neurosurgery Research Center, Research Institute of Functional Neurosurgery, Shohada Tajrish Comprehensive Neurosurgical Center of Excellence, Shahid Beheshti University of Medical Sciences, Tehran, Iran; e Centre of Excellence for Global Health (CEGH), Department of Global Health and Public Policy, School of Public Health, Tehran University of Medical Sciences, Tehran, Iran; f Neuroscience Research Center, Institute of Neuroscience and Cognition, Shahid Beheshti University of Medical Sciences, Tehran, Iran; g School of Pharmacy, Kerman University of Medical Sciences, Kerman, Iran

**Keywords:** Aminoguanidine, Arsenic toxicity, Oxidative stress, Hepatoprotection, Antioxidants

## Abstract

**Background:**

Arsenic exposure through environmental contamination poses significant public health concerns via oxidative stress-mediated toxicity. While aminoguanidine (AG) demonstrates antioxidant properties, its protective effects against arsenic-induced hepatotoxicity remain unexplored.

**Methods:**

Male mice (n = 32) were randomized into four groups (n = 8 per group): control (distilled water), arsenic (50 ppm sodium arsenite in drinking water), and two treatment groups receiving arsenic plus aminoguanidine (50 or 100 mg/kg/day, i.p.) for 28 days. Hepatic oxidative stress markers, plasma antioxidant capacity, and liver histopathology were evaluated.

**Results:**

Arsenic exposure induced significant liver histopathological changes (grade +2) and oxidative damage. AG treatment at 50 mg/kg/day showed optimal protective effects, with some samples displaying normal hepatic structure (grade 0) and others showing minimal changes (grade +1). This dose effectively reduced lipid peroxidation and protein carbonylation in liver tissue. The higher AG dose (100 mg/kg/day) demonstrated less protective effect, though it significantly improved plasma antioxidant capacity compared to the arsenic group.

**Conclusions:**

Aminoguanidine demonstrates dose-dependent hepatoprotective effects against arsenic-induced oxidative damage, with 50 mg/kg/day showing optimal efficacy. Further investigation of lower doses over extended periods is warranted to establish its therapeutic potential in arsenic toxicity.

## Highlights

• Aminoguanidine provides dose-dependent protection against arsenic-induced liver damage.
• 50 mg/kg optimal dose: 62.5 % normal histology, reduced oxidative stress markers.
• Inverted dose-response: 100 mg/kg less effective than 50 mg/kg.
• Multiple protective mechanisms: histological preservation and biochemical improvement.
• Novel therapeutic approach for arsenic toxicity with defined optimal window.

## Graphical Abstract

**Figure:**
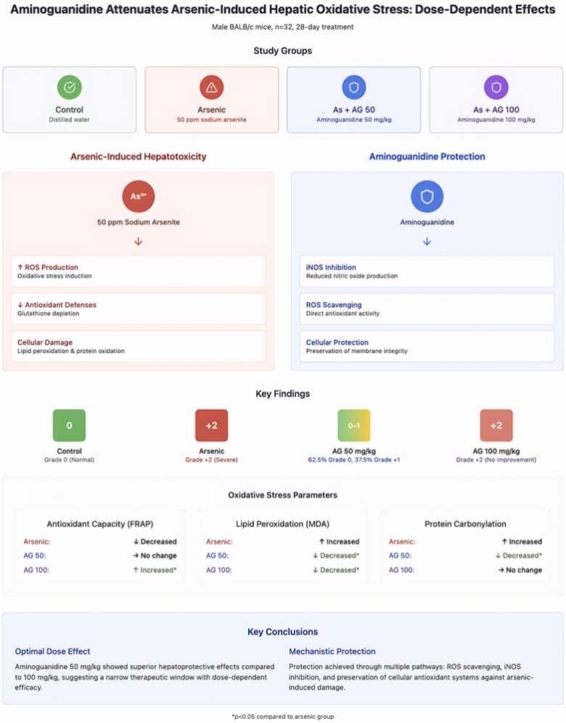

## 1 Introduction

Arsenic is a well-known toxic metalloid naturally present in the earth's biosphere. Environmental contamination occurs through both natural phenomena (leaching and runoff) and human activities, affecting populations worldwide [1]. Recent field studies demonstrate organ-specific bioaccumulation of heavy metals in naturally exposed animals, reinforcing the relevance of environmental toxicology research. Bruno et al. (2025) documented significant variation in mineral element levels among different organs of dogs exposed to volcanic pollution, providing evidence of metal bioaccumulation from chronic environmental exposure [2].

The toxicity of arsenic manifests through multiple mechanisms. Primary pathways include disruption of cellular energy metabolism through interference with the tricarboxylic acid cycle and ATP production, alongside the induction of oxidative stress [3], [4], [5]. Additionally, arsenic demonstrates a high affinity for sulfur and sulfhydryl groups, thereby inhibiting both enzymatic and non-enzymatic antioxidant systems [6]. This dual mechanism of increasing oxidative stress while simultaneously compromising cellular antioxidant defenses makes arsenic particularly toxic [1].

The liver's central role in arsenic metabolism makes it highly susceptible to toxic damage. Extended arsenic exposure triggers multiple hepatic complications, including oxidative damage, cell death and hepatocyte apoptosis. These effects are mediated through specific molecular pathways—JNK and p38 MAPK—which become activated by arsenic-mediated oxidative stress and contribute to progressive liver damage [7].

Aminoguanidine functions as a promising antioxidant compound against oxidative stress damage through its selective inhibition of inducible nitric oxide synthase (iNOS). This nucleophilic hydrazine compound has exhibited protective capabilities across multiple organs, with notable benefits for both hepatic and renal systems [8], [9]. Aminoguanidine's potential against arsenic toxicity is multifaceted. Beyond its known iNOS inhibition, it acts as a potent scavenger of reactive oxygen species, directly combating arsenical oxidative stress. It helps preserve cellular glutathione levels, crucial for maintaining antioxidant defenses compromised by arsenic [8]. Aminoguanidine also inhibits the formation of advanced glycation end-products, which may be relevant in arsenic-related metabolic disruptions [10]. Furthermore, its ability to modulate inflammatory responses through iNOS inhibition and antioxidant actions is particularly significant in mitigating arsenic-mediated organ damage, especially in the liver and kidneys, where chronic inflammation plays a key role [8]. These diverse mechanisms provide a strong rationale for investigating aminoguanidine as a potential therapeutic agent against arsenic toxicity [8], [11].

Previous antioxidant research against arsenic toxicity has explored several interventions: vitamins C and E, selenium and various plant compounds. These studies demonstrated effectiveness in reducing arsenic-induced oxidative damage and its subsequent health impacts [12], [13]. Aminoguanidine was selected for its distinctive dual function as both a selective inhibitor of inducible nitric oxide synthase (iNOS) and an effective scavenger of reactive oxygen and carbonyl species, providing broader cytoprotective coverage than classic antioxidants. Unlike vitamins C and E or selenium, aminoguanidine also inhibits advanced glycation end-product (AGE) formation and directly modulates inflammatory signaling, giving it therapeutic advantages in the context of arsenic-induced oxidative and nitrosative stress [8], [9], [10].

Current therapeutic options for subacute and chronic arsenic exposure remain limited, with no established safe and effective treatment protocol. However, emerging evidence from clinical and animal studies suggests that antioxidant supplementation may offer protection by preventing or slowing oxidative damage [14].

The existing research gap necessitates exploration of novel antioxidant interventions for arsenical toxicity. Key areas requiring investigation include their mechanistic pathways, dose-response relationships and efficacy in both preventing and potentially reversing oxidative damage from arsenic exposure [12], [15]. Determining the optimal dosage of antioxidant treatments is essential for developing effective arsenic toxicity protocols. Previous research demonstrates aminoguanidine's significant dose-dependent effects against free radical damage, indicating its potential efficacy as a therapeutic agent for arsenic-mediated oxidative stress [16].

This investigation seeks to evaluate aminoguanidine's dose-dependent effects on arsenic-induced hepatic oxidative stress using a mouse model. Based on aminoguanidine's established antioxidant mechanisms and iNOS inhibition properties [8], [16], we hypothesize that aminoguanidine administration will reduce liver damage and oxidative stress markers in arsenic-exposed mice. However, antioxidant dose-response relationships may be non-linear, with optimal protective effects occurring within specific therapeutic windows. We therefore anticipate that protective effects may vary between the tested doses, with intermediate doses potentially providing optimal efficacy.

## 2 Materials and methods

### 2.1 Experimental animals

Male BALB/c mice (20–40 g, 8–10 weeks old) were sourced from the Neuroscience Research Center, Kerman University of Medical Sciences, Kerman, Iran. At study onset, the mean initial body weight of the mice was 27.5 ± 2.0 g (mean ± SD), ensuring physiological comparability and minimizing variation in metabolism and toxicological sensitivity among experimental groups. Animals were housed under controlled laboratory conditions (12-hour light/dark cycle, 20°C, relative humidity 25–30 %) with ad libitum access to a standard rodent diet and water throughout the study period. All experimental procedures were approved by the Institutional Animal Ethics Committee of Kerman University of Medical Sciences (Ethics Code: IR.KMU.REC.1400.302) and conducted in accordance with international guidelines for laboratory animal care and use.

### 2.2 Experimental design

Thirty-two male mice were randomized into four experimental groups (n = 8/group):

1. Negative Control: Received distilled water
2. Positive Control: Received arsenic (50 ppm sodium arsenite in drinking water)
3. Treatment Group 1: Received arsenic (50 ppm) + aminoguanidine (50 mg/kg/day, i.p.)
4. Treatment Group 2: Received arsenic (50 ppm) + aminoguanidine (100 mg/kg/day, i.p.)

**Fig. 1:**
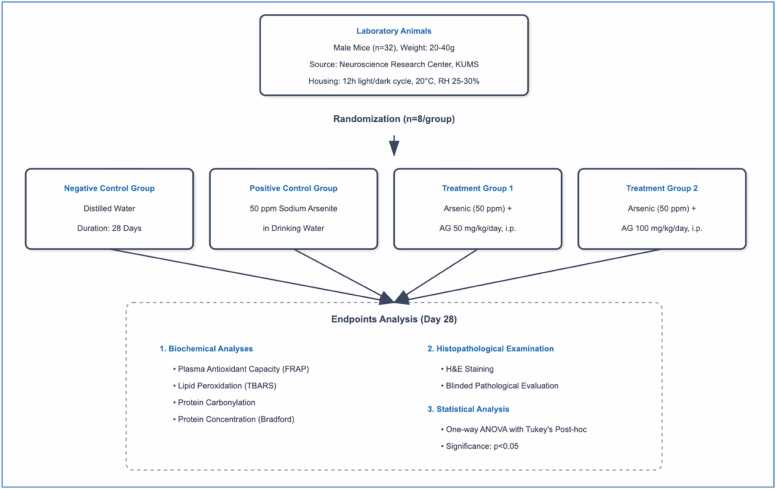
**Experimental design flowchart illustrating the 28-day study protocol.** Thirty-two male mice were randomly assigned to four groups (n = 8/group): negative control (distilled water), positive control (50 ppm sodium arsenite), and two aminoguanidine (AG) treatment groups receiving arsenic plus AG at 50 or 100 mg/kg/day intraperitoneally. The study endpoints included biochemical analyses of oxidative stress markers, histopathological examination, and statistical evaluation. FRAP: ferric reducing antioxidant power; TBARS: thiobarbituric acid reactive substances; H&E: hematoxylin and eosin.

Randomization was performed using a computer-generated random number sequence, ensuring unbiased and allocation-concealed assignment of animals to experimental groups. The treatment period was 28 days. Animals were monitored daily for clinical signs and body weight changes. At study completion, mice were anesthetized using a ketamine/xylazine combination. Blood and liver samples were collected for subsequent analyses. The experimental design is illustrated in Fig. 1.

### 2.3 Dose selection rationale

The arsenic dose (50 ppm sodium arsenite in drinking water) was selected based on established protocols demonstrating hepatic oxidative stress in mouse models [1], [4], [17]. Aminoguanidine doses (50 and 100 mg/kg, i.p.) were selected based on literature demonstrating optimal antioxidant and organ-protective effects within this therapeutic range [10], [18], [19], [20].

### 2.4 Sample collection and processing

At the end of the 28-day treatment period, animals were fasted for 12 h and then anesthetized using an i.p. injection of ketamine (90 mg/kg) and xylazine (10 mg/kg) administered as a single injection. If additional anesthesia was needed, supplemental ketamine was administered at one-third of the initial ketamine dose. Throughout the procedure, body temperature was maintained using a heating pad and depth of anesthesia was confirmed by testing for absence of pedal withdrawal and corneal reflexes. Blood samples were collected via cardiac puncture into EDTA-containing tubes and centrifuged at 3000 rpm for 15 min at 4°C to obtain plasma. Liver tissues were immediately excised, rinsed with ice-cold saline and divided into portions for biochemical analysis (stored at −80°C) and histopathological examination (fixed in 10 % neutral buffered formalin).

### 2.5 Biochemical analyses

#### 2.5.1 Plasma antioxidant capacity assay

Total antioxidant capacity was evaluated using the ferric reducing antioxidant power (FRAP) assay. The method is based on the reduction of the ferric-tripyridyltriazine complex to its ferrous form under acidic conditions, producing a blue chromogen measurable at 593 nm [21], [22].

#### 2.5.2 Lipid peroxidation assay

Tissue malondialdehyde (MDA) levels were quantified as a marker of lipid peroxidation using the thiobarbituric acid reactive substances (TBARS) method [23]. Results were expressed as nmol MDA/mg protein. A standard curve was constructed using serial dilutions of 1,1,3,3-tetramethoxypropane (TMP) as MDA equivalent standards ranging from 0 to 10 μM. The linear regression equation and correlation coefficient (r²) of the standard curve were determined to validate assay linearity (Figure 3–2: The standard curve of TBARs is shown in the following figure).

#### 2.5.3 Protein carbonylation assay

Protein oxidation was assessed by measuring carbonyl groups according to Levin's method. The assay involves the reaction of 2,4-dinitrophenylhydrazine with protein carbonyl groups, forming a Schiff base that produces a quantifiable yellow complex (absorbance at 370 nm) [24], [25].

#### 2.5.4 Protein concentration assay

Protein concentrations were measured using the Bradford method, based on the binding of Coomassie Brilliant Blue dye to proteins, resulting in a colorimetric change measurable spectrophotometrically [26], [27]. (Figure 3–3: The standard Bradford curve is shown below.)

### 2.6 Histopathological examination

Liver tissue samples were fixed in buffered formalin, processed through standard protocol and stained with hematoxylin and eosin (H&E) for microscopic examination. Histopathological changes were evaluated by a pathologist blinded to the treatment groups.

### 2.7 Statistical analysis

Sample size (n = 8 per group) was determined based on previous similar studies and pilot experiments. Data analysis was performed using GraphPad Prism version 9.0 software. Data normality was assessed using the Shapiro-Wilk test. Results are expressed as Mean ± SD. Statistical comparisons between groups were conducted using one-way ANOVA followed by Tukey's multiple comparison test for normally distributed data. For histopathological data (categorical), the chi-square test was used. All biochemical analyses were performed in duplicate. Statistical significance was set at p < 0.05.

## 3 Results

### 3.1 Histopathological findings

Control group liver tissue demonstrated normal hepatic architecture with proper hepatocyte organization, centrally positioned nuclei and normal cytoplasmic appearance (Figs. 2–1).

**Fig. 2.1:**
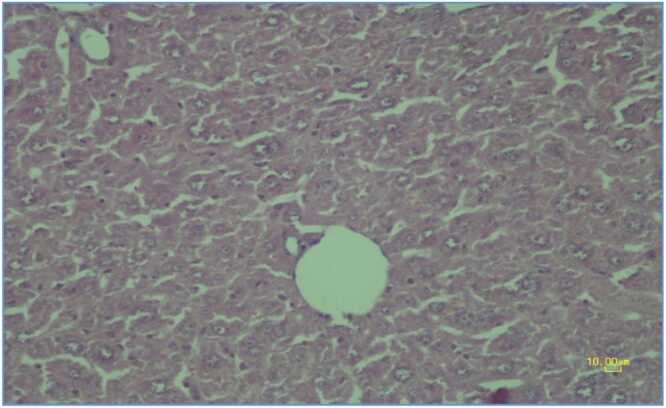
**Control Group**. Photomicrograph of liver tissue in the control group demonstrating normal hepatic architecture with proper hepatocyte organization, centrally positioned nuclei and normal cytoplasmic appearance. Normal liver tissue stained with hematoxylin-eosin × 200, scale bar: 10.00 μm.

Both the arsenic group and the arsenic plus aminoguanidine 100 mg/kg group showed moderate grade (+2) degenerative changes on H&E staining. These changes were characterized by significant hepatocyte cytoplasmic vacuolation, cell swelling and sinusoidal fading. The severity of tissue damage was similar between these two groups (Fig. 2–2). Histopathological changes did not differ significantly between the aminoguanidine 100 mg/kg and arsenic groups (p > 0.05, chi-square test).

**Fig. 2.2:**
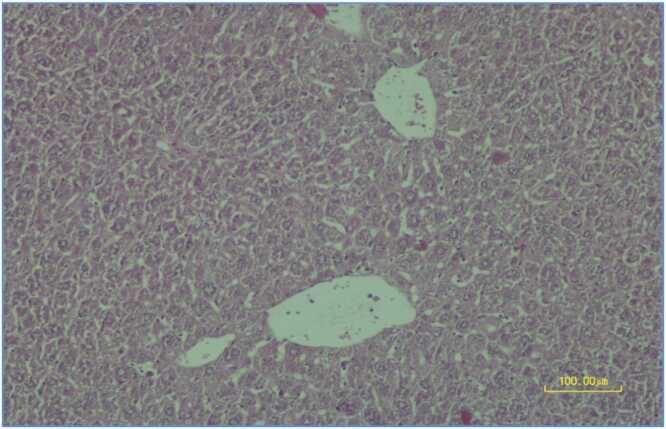
**Arsenic Group.** Photomicrograph showing moderate degenerative changes (grade +2) in hepatocytes characterized by significant cytoplasmic vacuolation, cell swelling and sinusoidal fading. Liver section from the arsenic-treated group stained with hematoxylin-eosin, scale bar: 100.00 μm.

The arsenic plus aminoguanidine 50 mg/kg group showed variable but improved responses in liver histology. Five out of eight animals (62.5 %) exhibited normal hepatocyte structure (grade 0) (Fig. 2–3), while three out of eight animals (37.5 %) showed mild degenerative changes (grade +1) characterized by minimal hepatocyte cytoplasmic vacuolation and cell swelling (Figs. 2–4). No animals in this group showed grade + 2 changes.

**Fig. 2.3:**
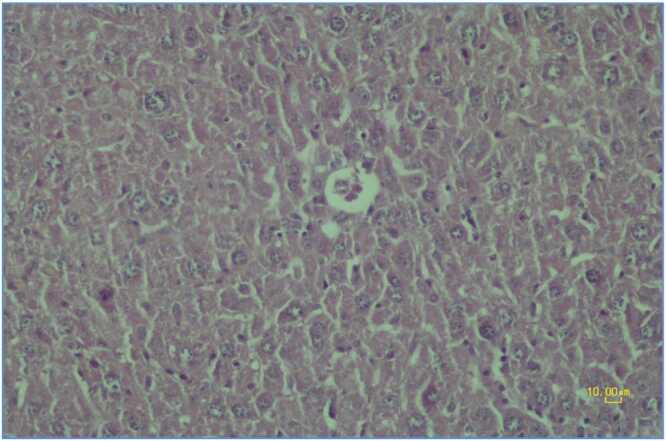
**Arsenic plus Aminoguanidine (50 mg/kg) Group.** Photomicrograph of a liver section exhibiting mild degenerative changes in hepatocytes with the formation of small vacuoles in the cytoplasm. Hematoxylin-eosin staining, scale bar: 10.00 μm.

**Fig. 2.4:**
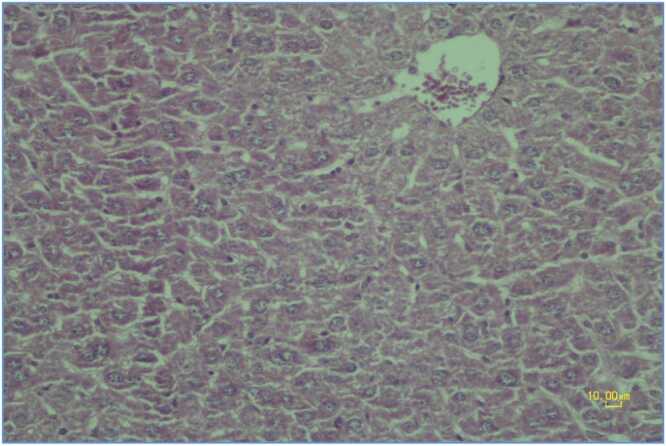
**Arsenic plus Aminoguanidine (100 mg/kg) Group.** Photomicrograph showing hepatocytes with eosinophilic cytoplasm and vesicular nucleus, demonstrating moderate degenerative changes (grade +2) similar to the arsenic-only group. Hematoxylin-eosin staining, scale bar: 10.00 μm.

**Table 1:** Histopathological Grading of Liver Tissue in Different Treatment Groups.

**Group**	Histopathological Grade*
Control	Grade 0
Arsenic (50 ppm)	Grade + 2
Arsenic + AG (50 mg/kg)	Grade 0–1**
Arsenic + AG (100 mg/kg)	Grade + 2

*Grading criteria:

- Grade 0: Normal hepatic architecture

- Grade + 1: Mild degenerative changes with minimal hepatocyte cytoplasmic vacuolation

- Grade + 2: Moderate degenerative changes with significant hepatocyte cytoplasmic vacuolation and cell swelling

**Two samples showed Grade 0 (normal structure), and one sample showed Grade + 1 (mild changes).

### 3.2 Oxidative stress parameters

#### 3.2.1 Results on total antioxidant capacity of plasma

Plasma total antioxidant capacity was significantly decreased in the arsenic group compared to the control group. Among treatment groups, only the aminoguanidine 100 mg/kg group showed a significant difference when compared to the arsenic group (Figs. 3–1).

**Fig. 3.1:**
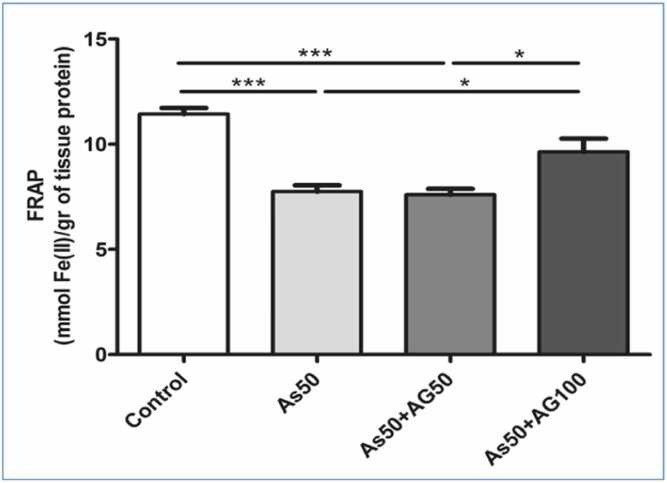
**Ferric Reducing Antioxidant Power Graph.** FRAP (Ferric Reducing Antioxidant Power) levels in tissue show reduced antioxidant capacity in arsenic-treated groups (As50) compared to control, with partial recovery in aminoguanidine treatment groups (As50 +AG50 and As50 +AG100). Statistical significance indicated by: *p < 0.05, **p < 0.01, ***p < 0.001 compared to the control group; †p < 0.05, ††p < 0.01, †††p < 0.001 compared to the arsenic group.

#### 3.2.2 Results on lipid peroxidation on liver tissue

Hepatic lipid peroxidation analysis revealed a significant increase in the arsenic group compared to controls. Both aminoguanidine doses (50 and 100 mg/kg) effectively reduced lipid peroxidation levels compared to the arsenic group, with significant differences observed at both doses (Fig. 3–2).

**Fig. 3.2:**
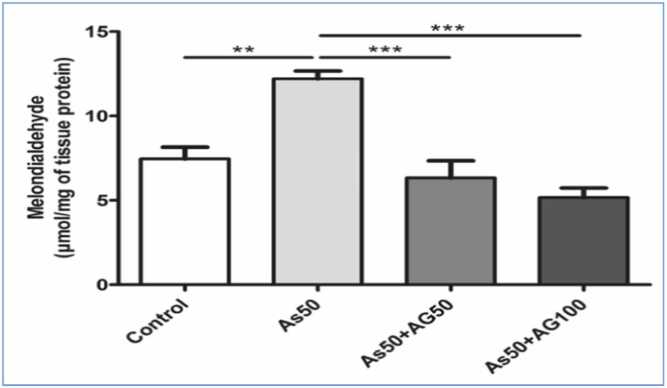
***Malondialdehyde Assessment Graph.*** Malondialdehyde levels in tissue as a marker of lipid peroxidation, demonstrating significant elevation in the arsenic-treated group (As50) compared to control and amelioration with aminoguanidine treatment (As50 +AG50 and As50 +AG100). Statistical notation as in Fig. 3.1.

### 3.3 Effects of carbonyl protein on liver tissue

Assessment of protein oxidation showed significantly increased carbonyl content in the arsenic group compared to controls. A significant reduction in protein carbonylation was observed in the arsenic plus aminoguanidine 50 mg/kg group compared to the arsenic group (Fig. 3–3). To illustrate the relationships between different oxidative stress parameters, we created a conceptual summary of all measured biochemical markers (Fig. 4). This Figure represents a conceptual model only; no multivariate statistical analyses were performed. The results demonstrate that arsenic exposure led to a concurrent decrease in antioxidant capacity and increases in both lipid peroxidation and protein oxidation markers. Aminoguanidine treatment at 50 mg/kg showed the most balanced protective effect across all parameters, while the higher dose (100 mg/kg) showed differential effects on various markers.

**Fig. 3.3:**
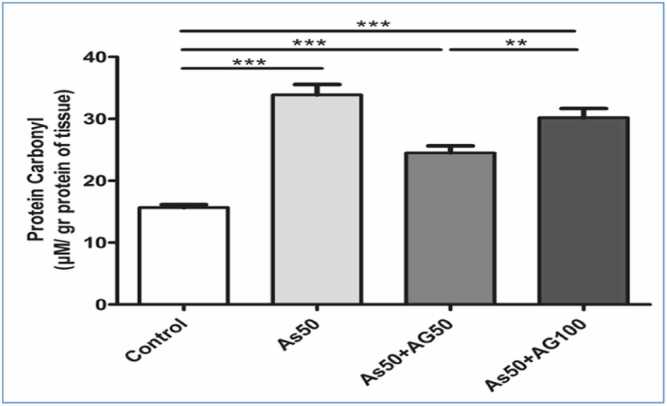
**Protein Carbonyl Content Graph.** Protein carbonyl content in tissue showing oxidative protein damage. Arsenic treatment (As50) significantly increased carbonyl levels compared to control, while aminoguanidine co-treatment (As50 +AG50 and As50 +AG100) partially reduced protein oxidation. Statistical notation as in Fig. 3.1.

**Fig. 4:**
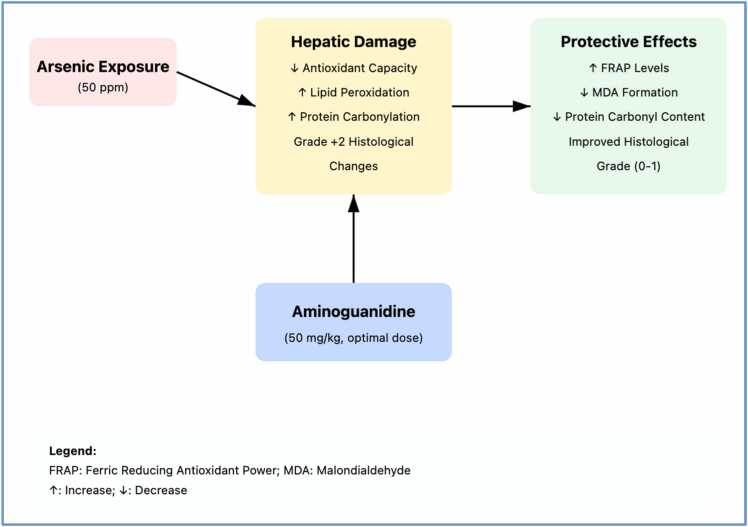
**Proposed mechanisms of aminoguanidine protection against arsenic-induced hepatotoxicity**. Arsenic exposure (50 ppm) induces hepatic damage characterized by decreased antioxidant capacity, increased lipid peroxidation, elevated protein carbonylation, and grade + 2 histological changes. Aminoguanidine treatment (50 mg/kg) provides optimal protection by enhancing antioxidant defenses (increased FRAP), reducing oxidative damage (decreased MDA and protein carbonyl content), and improving histological grade (0−1). Note: Fig. 4 is a conceptual schematic and not based on multivariate statistical analysis.

## 4 Discussion

This study demonstrates that aminoguanidine provides significant hepatoprotection against arsenic-induced oxidative injury, with 50 mg/kg emerging as the optimal dose. The findings indicate that this dose effectively counteracts arsenic-induced oxidative damage through enhancement of antioxidants capacity and reduction of both lipid and protein oxidation.

Our results confirm arsenic exposure (50 ppm for 28 days) induced substantial hepatic damage characterized by grade + 2 histopathological changes, decreased total antioxidant capacity, increased lipid peroxidation, and elevated protein carbonylation. These findings are consistent with previous observations demonstrating arsenic's ability to reduce antioxidant capacity [28] and increase lipid peroxidation [29], [30]. Histopathological examination confirmed the liver as a major target organ for arsenic toxicity, likely reflecting its central role in xenobiotic biotransformation and detoxification. The most significant finding is the non-linear dose-response relationship observed with aminoguanidine treatment. Notably, aminoguanidine at 100 mg/kg provided less histopathological protection than the 50 mg/kg dose, indicating a non-linear dose-response relationship. This inverted dose-response pattern suggests potential pro-oxidant effects at higher concentrations or saturation of protective pathways, emphasizing the importance of dose optimization. Similar U-shaped dose-response relationships have been documented for other antioxidants [31]. The 50 mg/kg dose achieved remarkable hepatoprotection, with 62.5 % of animals showing normal hepatic architecture (grade 0) and the remainder exhibiting only mild changes (grade +1). The protective effects at 50 mg/kg against both lipid peroxidation and protein carbonylation indicate comprehensive cellular protection against oxidative damage. In contrast, the higher dose failed to provide significant histopathological improvement despite some biochemical benefits, suggesting that higher concentrations may interfere with normal cellular functions or activate alternative pathways [32]. Arsenic toxicity involves excessive ROS production and disruption of endogenous antioxidant systems [33]. This elevated ROS production leads to oxidative damage of cellular components, including membrane lipids, proteins, enzymes, and nucleic acids, resulting in increased TBARS content, protein carbonylation, and DNA fragmentation [34], [35]. Lipid peroxidation, a primary consequence of free radical damage, compromises cellular membrane integrity and function, ultimately promoting cell necrosis [36]. ROS-mediated cell death occurs through both internal (mitochondrial) and external (death receptor) pathways [35]. Aminoguanidine's protective mechanisms likely involve its established properties: selective inhibition of inducible nitric oxide synthase (iNOS), direct scavenging of reactive oxygen species, and inhibition of advanced glycation end-product formation. The compound's multifaceted approach may provide broader cytoprotective coverage compared to traditional antioxidants. Previous research has established aminoguanidine's antioxidant properties, demonstrating its ability to enhance antioxidant enzyme activity and prevent oxidative and nitrosative cell damage [37], [38]. This protection is particularly relevant given oxidative stress's role in various pathologies, including cardiovascular disease, diabetes, cancer, and Alzheimer's disease [39], [40]. Previous studies have expanded understanding of aminoguanidine's therapeutic potential, showing protection against cisplatin-induced nephrotoxicity and radiation-induced lung toxicity. Notably, Abdel-Zaher et al. demonstrated aminoguanidine's ability to preserve liver and kidney antioxidant stores during acetaminophen exposure [40], [41]. Arsenic's environmental impact extends beyond hepatotoxicity, causing both acute and chronic toxicity in multiple organs, including skin, liver, and kidney, with associated cancer risks through oxidative damage mechanisms [42], [43]. These findings contribute to addressing the limited therapeutic options available for arsenic toxicity, where current treatment protocols remain inadequate. To illustrate the relationships between different oxidative stress parameters observed in this study, we created a conceptual summary of all measured biochemical markers (Fig. 4). This figure represents a conceptual model only; no multivariate statistical analyses were performed. The framework demonstrates that arsenic exposure leads to concurrent decreases in antioxidant capacity and increases in both lipid peroxidation and protein oxidation markers, while aminoguanidine treatment at 50 mg/kg provides balanced protective effects across all parameters.

### 4.1 Study limitations

Several limitations of the present study warrant acknowledgment. The study design presents certain constraints: First, the 28-day treatment duration, while sufficient to demonstrate acute protective effects, may not reflect long-term chronic arsenic exposure scenarios relevant to human populations. Second, the study was conducted exclusively in male mice, which may limit the generalizability of findings to female subjects. Third, The relatively small sample size (n = 8 per group) may limit the statistical power and generalizability of our findings. From a methodological perspective, all biochemical analyses were performed in duplicate rather than triplicate, which may reduce the ability to identify outliers and could affect data accuracy. The study did not include an ascorbic acid-only (vitamin C) treatment group, which could have served as an additional antioxidant comparator to better contextualize aminoguanidine's protective effects. Mechanistically, the protective effects of aminoguanidine were inferred from biochemical and histological outcomes rather than direct mechanistic investigations. Additionally, the lack of measurement of biomarkers beyond oxidative stress markers, such as hepatic iNOS activity, limits mechanistic insights into aminoguanidine’s mode of action against arsenic toxicity. Finally, the optimal dose range requires further characterization, particularly given the unexpected superiority of the lower dose tested, which suggests the need for more comprehensive dose-response studies to establish the therapeutic window for aminoguanidine's hepatoprotective effects.

## 5 Conclusions

This study demonstrates that aminoguanidine provides hepatoprotection with a non-linear dose-response relationship against arsenic-induced oxidative injury, with 50 mg/kg emerging as the most effective dose. The findings highlight a narrow therapeutic window, as higher doses may not offer additional protection and can even diminish beneficial outcomes. Further research should clarify mechanistic pathways, establish optimal dosing strategies and evaluate translational potential in clinical settings.

## CRediT authorship contribution statement

**Behnam Ghorbani-Nejad:** Writing – review & editing, Writing – original draft, Formal analysis. **Matin Baghani:** Writing – review & editing, Writing – original draft, Visualization. **Leila Poudineh:** Writing – original draft. **Somayyeh Karami-Mohajeri:** Writing – review & editing, Supervision. **Nastaran Allahdini Hasaruyieh:** Writing – original draft. **Mahshid Jamshidi:** Writing – review & editing, Writing – original draft. **Milad Rahimzadegan:** Writing – review & editing, Supervision. **Jafar Ahmadi:** Writing – original draft.

## Declaration of Competing Interest

The authors declare that they have no known competing financial interests or personal relationships that could have appeared to influence the work reported in this paper.

## Data Availability

Data will be made available on request.
